# Protocols for sample preparation and compound-specific stable-isotope analyses (δ^2^H, δ^13^C) of fatty acids in biological and environmental samples

**DOI:** 10.1016/j.mex.2023.102283

**Published:** 2023-07-08

**Authors:** Matthias Pilecky, Leonard I. Wassenaar, Sami Taipale, Martin J. Kainz

**Affiliations:** a WasserCluster Biologische Station Lunz, Inter-University Center for Aquatic Ecosystem Research, Dr. Carl-Kupelwieser Promenade 5, 3293 Lunz/See, Austria; b Research lab of Aquatic Ecosystem Research and -Health, Danube University Krems, 3500 Krems, Austria; c University of Jyväskylä, Department of Biological and Environmental Science, Survontie 9C, Finland

**Keywords:** Carbon isotopes, Deuterium, Fatty acids, GC-IRMS, Stable-isotopes, CSIA, Reproducible determinations of the δ^2^H and δ^13^C composition of fatty acids from environmental samples.

## Abstract

Compound-specific stable-isotope analysis (CSIA) of fatty acids is a powerful tool to better understand the trophic transfer of fatty acids and their biochemical fate in and across ecosystems, including tracing animal migration and understanding physiological processes. The non-exchangeable nature of C—H bonds in acyl chains, hydrogen (*δ*^2^H) and carbon (*δ*^13^C) stable-isotope values of fatty acids (FA) provide independent information about the origins of fatty acids. Several technical obstacles must be overcome to ensure accurate and reproducible measurements of FA-CSIA can be made. This protocol describes the sample preparation process for successful stable-isotope analyses of fatty acids obtained from environmental and biological samples. Numerous techniques for the preanalytical processing of fatty acid samples are available, and these often have minimal impact on *δ* values. Here, we provide an in-depth guide detailing our well-established laboratory protocols, ranging from the initial sample preparation, lipid extraction, and transmethylation to the instrumental arrangement, data collection, and analysis.

• Protocol from obtaining a sample to standardized fatty acid specific *δ*^2^H and *δ*^13^C values.
• Separate GC analysis procedures for C and H are recommended for optimal performance.

## Graphical abstract

**Figure:**
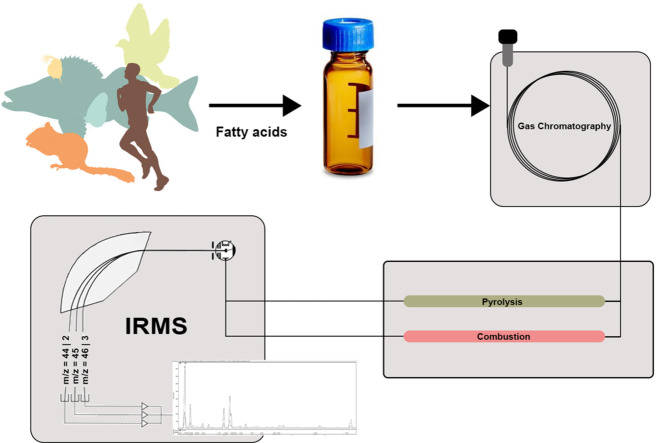

Specifications table

**Table:** 

Subject area:	Environmental Science
More specific subject area:	*δ*^2^H and *δ*^13^C Isotope Analysis of lipid components
Name of your method:	Reproducible determinations of the *δ*^2^H and *δ*^13^C composition of fatty acids from environmental samples.
Name and reference of original method:	New Method
Resource availability:	N/A

**Method details**

## Introduction

Bulk stable-isotope analysis (e.g., *δ*^13^C, *δ*^2^H) of primary producers and consumer tissues (e.g., algae, leaves, feathers, muscle tissue, etc.) has been used since the 1980s to construct trophic models for terrestrial, freshwater, and marine ecosystems [1], [2], [3], [4], [5]. However, bulk sample stable-isotope analysis suffers from several inherent challenges: (i) bulk tissue isotopic averaging of all molecular components, such as nucleic acids, carbohydrates, proteins, and lipids, all of which undergo continual anabolic and catabolic processes at different turnover rates, potentially distorting the bulk *δ*^2^H or *δ*^13^C value depending on the current physiological state of the biological sample; (ii) H or C stable isotopic fractionation by biochemical processes, either in vivo or during sample preparation and analysis; (iii) overlap of bulk stable-isotope values among available food sources, and; (iv) in case of *δ*^2^H, hydrogen isotope exchange with environmental water [6], [7], [8]. In the past decade, interest in compound-specific stable-isotope analyses (CSIA) via the study of the isotopic composition of specific molecules (e.g., essential or non-essential amino acids) extracted from bulk or specific tissues arose with hopes of gaining a deeper understanding of species interactions in trophic ecology in general, and to unravel ecophysiological processes in particular. In contrast to bulk stable isotope analyses, CSIA requires specialized sample preparation and complex gas or liquid chromatographic (GC/LC) interfaces to separate and combust or reduce the individual molecules for analysis.

One group of molecules for which CSIA is particularly important in trophic ecology are fatty acids (FA). Physiologically, FA are used as an energy source, usually stored in neutral lipids (NL), but also comprise the central building block of biological membranes in the form of polar lipids (PL) [9]. Furthermore, some molecular FA species are a synthesis platform for bioactive signaling molecules [10], [11], [12]. Quantitative compositional analysis of FA has been used to assess dietary composition [13], as well as the trophic transfer of specific dietary energy sources, such as bacteria, algae, or terrestrial-derived fatty acids [14], [15], [16]. However, it is problematic to differentiate amongst different dietary sources of fatty acids based on composition alone; for example, the essential polyunsaturated fatty acids (PUFA) alpha-linolenic (ALA) and linoleic (LIN) acids cannot be synthesized by consumers but are synthesized in both terrestrial and aquatic primary producers [17], and hence cannot be used as distinct dietary source markers. This problem can be circumvented by applying CSIA because *δ*^2^H and *δ*^13^C values of FA often correlate with the stable-isotopic composition of ambient hydrogen and carbon sources, such as water and food, but are subjected to molecular – and often taxonomic species-specific – stable isotopic fractionation [18]. Once FA are formed, the C—H bond of the alkyl chains is chemically stable and non-exchangeable with ambient water, preserving the original H isotope source signal at contemporary timescales [19]. When transferred to aquatic or terrestrial consumer tissues, the general idea is that the *δ*^2^H and *δ*^13^C values of essential FA (i.e., LIN and ALA) reflect their direct dietary sources of consumers.

In contrast, the non-essential FA compounds provide information about metabolic processes [20], and their *δ*^13^C values correlate with primary energy sources, while their *δ*^2^H values correlate with ambient water [21]. It has also been shown that the FA composition of NL closely reflects dietary sources, whereas, the composition of PL is mainly genetically determined and more independent of dietary input [22], although alterations due to nutritional limitations are known [9]. Thus, it might be helpful for specific study settings to perform lipid class-specific FA-CSIA.

In different study settings, we recently demonstrated that carbon and hydrogen isotope values of fatty acid generally show independent patterns [21,[23], [24], [25], [26], [27]]; however, these patterns might influence each other and exhibit negative kinetic inhibition in metabolic processes if one isotope is artificially enriched in an experimental setup [21]. So far, our findings also indicate that *δ*^13^C values of FA are conservative, meaning they show fewer inter-species and metabolic (i.e., ecophysiological) dynamics within a specific spatial and temporal area and have a high potential for particular site tracing of the origin of a sample. In contrast, *δ*^2^H values of FA are highly sensitive to environmental and physiological changes. They can exhibit a comparatively extensive range of *δ*-values; thus, H isotopes are preferable when investigating metabolic and ecophysiological processes [21,25].

In this context, it should be noted that lipid synthesis leads to significant ^2^H depletion (on average 150 ‰) in the synthesized organic matter. Yet, environmental water and lipid *δ*^2^H values in primary producers are usually strongly correlated [28,29]. The ^2^H depletion can be partially explained by H-isotopic fractionation due to recurrent elongation and desaturation [30], but is also strongly dependent on the physiological processes of the individual species. When comparing *δ*^2^H values of fatty acids between diets and consumers, four key processes with high potential for H-isotope fractionation have to be considered: (1) H-isotope fractionation during dietary fatty acid uptake and allocation to individual tissues; (2) the H-isotope composition of cellular water, acetate, and NADPH, which are used for fatty acid synthesis and modification; (3) H-isotope fractionation by enzymes involved in the lipid biosynthetic pathways, which in general utilize lighter substrates faster than their heavier isotopologues, and; (4) H-isotope fractionation due to fatty acid transport and metabolism [31]. As a result, fatty acids that a consumer synthesize are often ^2^H-depleted compared to the diet, while fatty acids that are not needed by a consumer and thus catabolized become comparatively enriched in ^2^H [24,25].

Here, we describe a detailed analytical protocol for reliable determinations of the *δ*^2^H and *δ*^13^C values of FA in environmental samples. We acknowledge this protocol is not unique, and other protocols (e.g., lipid extraction, GC setup, and GC columns) and instrumentation may produce similar accurate results. We describe configurations and technical difficulties, including those that do not work, and our solutions to these problems, which could be adopted in other laboratories for troubleshooting specific issues, and should help new users who want to establish CSIA of FA in their lab.

## Considerations for study design and workflow of sample analysis

When using FA-CSIA in a study, the first consideration is that sufficient material can be collected during the planning design. As a rule of thumb, we recommend sampling enough material to obtain at least 0.4–0.5 mg of total lipid extract if doing both *δ*^2^H- and *δ*^13^C—CSIA, as described previously [1]. If only *δ*^13^C values are desired, 0.2–0.3 mg can suffice. In practice, for both isotopes, this translates to approximately 25 mg of dry sample weight (dw) for leaves (∼1–2 % lipid content), 5–15 mg dw for algal cultures, seston or biofilm (ca. 3–10 % lipid content), 2–3 mg dw for most invertebrates and muscle tissue (ca. 15–25 % lipid content), or 1–2 mg for lipid-rich tissue like liver or brain. The recommended minimum sample amount increases by 5-fold if polar and neutral lipids are to be separated from the total lipid extract for lipid class FA-CSIA. Finally, the amounts of "sufficient'' biomass noted above are general recommendations subject to sample type and the FA of interest. When studying ecophysiological processes, the relative amount of a target FA in samples could be low, which increases the minimal biomass required. If in doubt, it is better to extract lipids from larger samples as we have not observed any isotope-fractionation effects due to sample dilution or splitting (results not published). At the same time, signal amplitudes at the lower end of the instrument detection limits might also adversely affect *δ*^13^C and especially *δ*^2^H values.

In this protocol, the CSIA-FA workflow was divided into six steps, starting from freshly obtained samples to obtaining the final ^2^H or ^13^C stable-isotope results:

• Step 1: Sample storage, preservation, and preparation for the FA extraction procedure
• Step 2: Extraction of total lipids
• Step 2a (optional): Separation of the lipid classes
• Step 3: Transmethylation of FA
• Step 4a: Measuring by GC—C-IRMS for *δ*^13^C—CSIA
• Step 4b: Measuring by GC-P-IRMS for *δ*^2^H—CSIA
• Step 5: Data analysis
• Step 6: standardization and methanol isotope-corrections

Finally, we discuss some procedures for quality assurance and control (QA/QC) that might give the practitioner some clues about random and systematic errors, as well as instrumental drift correction.

## Step 1: sample preparation

Most environmental samples (organics, tissues, etc.) for FA-CSIA should be stored at -80 °C and ideally freeze-dried as quickly as possible. Samples can then be stored in sealed containers in dry conditions (e.g., desiccator) at room temperature for further processing. Before opening sample containers, frozen samples should be brought to room temperature to avoid moisture accumulation. Ideally, samples are homogenized to a fine powder using a glass mortar rod or stainless steel mill. The samples are weighed and transferred into a pre-baked glass test tube, i.e., that has been heated at 450 °C for at least two hours. Although for CSIA, the exact sample mass is of lesser importance, one can use the same extraction for quantitative FA analysis or assessment of total lipid content. An analytical microbalance (± 0.01 mg) is recommended in this case. Because multiple sample transfers of precise weights without material loss are unavoidable, sample containers are first tared, followed by gentle transfer of the required biomass into the glass tubes and using the mass difference from the initial sample containers as the dry weight reference. If homogenization of the sample is not possible, an alternative method is to cover samples with pure chloroform after recording the weight (the volume of chloroform used should also be documented) and to store the samples and solvent at −80 °C until the chloroform is completely frozen. This procedure ensures solvent exposure to the sample and facilitates the complete extraction of lipids. Furthermore, FA samples can be stored frozen with solvent for a prolonged time without sample degradation [32].

## Optional: use of internal standards and combination with quantitative FA analysis

In practice, quantitative analysis of FA (qFA) (e.g., via GC-FID or GC–MS) and FA-CSIA are done on the same sample extract. Some GC setups (e.g., using split valves) theoretically allow or simultaneously use multiple detectors for the same sample run. However, we recommend performing qFA and FA-CSIA each in a separate run because, from our experience, (i) GC-setups using splits tend to leak more often, requiring higher maintenance rates, introducing bias due to shifts in split flow rates, and leading to loss of sample, while, (ii) usually at least some FA peaks of interest tend to be on the lower end of peak height and area, and every bit of eluting component is needed to achieve acceptable detection using FA-CSIA.

Using the same sample for qFA also means an internal standard is required to account for (variable) sample recovery/loss during the pre-analytical procedures. Although an internal standard is not needed for FA-CSIA, internal standards help control system stability and instrumental drift correction. While many FA (e.g., 17:0, 19:0, ^13^C-18:0, 23:0) in free form or as PL and NL are described in the literature for qFA, we recommend using a form of 23:0 (free FA or esterified form of NL/PL) because 23:0 rarely occurs in natural samples. At the same time, GC peaks in FA-CSIA are much broader than in qFA, and an acceptable return to IRMS background takes much longer. Many FA used as internal standards interfere with other sample peaks, especially in complex matrices, complicating the data post-processing, if not making it impossible. For example, in many setups, 19:0 elutes close to alpha-linoleic acid (18:3n-3, ALA), gamma-linoleic acid (18:3n-6, GLA) or linoleic acid (18:2n-6, LIN), which are usually FA of ecological interest. In our experience, for most samples, there is a large gap between eicosapentaenoic acid (20:5n-3, EPA) peaks and the elution of FA such as 22:3–22:6/24:0/24:1. Within this gap, the internal standard 23:0 elutes, making it an ideal internal standard for FA-CSIA.

## Step 2: lipid extraction

Several methods are available to extract lipids. These include methods by; (i) Bloor [33], (ii) Folch, Lees, and Stanley [34], (iii) Bligh and Dyer [35], the chloroform-free method (iv) by Soxhlet using hot petrol-ether [36], which is time-consuming, (v) Smedes [37], and (vi) the BUME method [38]. Several studies compared these different extraction methods regarding efficiency for total lipids and individual lipid classes (e.g., see [38,39]). Bias in extraction efficiency of certain lipid classes could induce undesirable C- or H- isotope fractionation, i.e., differences in *δ*^2^H and *δ*^13^C values when performing lipid extraction for bulk analysis, either for the lipid extract or the remaining lipid-extracted sample [40]. While recovery impacts have not been evaluated for FA-CSIA, it can be argued that changes in the mass fractions of specific components could induce compositional bias. However, stable-isotope fractionation due to different extraction efficiencies of isotopomers and isotopologues is likely to be below analytical detection limits. Thus, while the choice of lipid extraction method might play a role in qFA, it is unlikely to play any significant role in FA-CSIA.

From our experience, we prefer the Folch [34] lipid extraction method using a chloroform:methanol:water (8:4:3 vol/vol) mix. The solvent mix volumes can be adjusted depending on the sample type, but at least 3 mL of organic solvents is recommended. If samples have been frozen with chloroform (above), add half the volume of the methanol mix. Otherwise, using a prepared mixture of chloroform: and methanol (2:1) submerge the sample. A 1/4^th^ of the volume of organic solvents used of deionized water is added to achieve the final ratio. Subsequently, sonicate the samples for 10 min, vortex them at max speed for 1 min, and centrifuge at 2000 *g* for 5 min. Next, the lower organic phase has to be transferred into a new glass tube using glass Pasteur pipettes, ideally without moving any particles (although a second purification process will follow, and a tiny amount of small particles is not as critical at this point). It is helpful not to fully squeeze the pipette before insertion, but squeeze halfway to gently increase the pressure when going through the upper aqueous phase. This way, a few expelled air bubbles prevent particles from entering the pipette without mixing the separated phases. However, this requires practice. To ensure extraction of all lipids, especially if the amount of biomass is limited, it is recommended to wash the pipette with 2 mL of chloroform, repeat the steps from sonification onwards 1–2 times, and pool all the organic phases. The extraction efficiency, however, does not affect the compound-specific isotope values.

If lipid class separation is performed, the combined organic phases are dried to approximately 0.5 mL using a gentle stream of inert gas (e.g., N_2_, Ar) and continue to Step 2a. This step helps purify complex matrixes with many interfering lipophilic substances that complicate the chromatograms, as can be seen frequently, e.g., in aquatic seston samples. If step 2a is skipped, the organic phases are completely dried using inert gas flow after adding 1 mL toluene. After this step, the procedure can be stopped, and samples stored at −80 °C until further processing.

## Step 2a: lipid class separation (optional)

For quantitative lipid class separation, we recommend the method of Kaluzny et al. [41], e.g., using solid phase extraction (SPE) by Bond Elut LRC – SI (Agilent Technologies, Santa Clara, CA). This method allows the separation of polar lipids (PL) and free fatty acids (FFA) from the bulk lipid extract (neutral lipids, NL), which can be further separated into mono/di/triglycerides, cholesterol and cholesteryl esters using a second cartridge if desired. For most FA-CSIA applications, FA analysis from PL and NL will be sufficient.

1. Condition the SPE columns using 4 mL hexane, then load the sample extract (maximum 0.5 mL or 10 mg of total lipid).
2. **NL:** Elute with 4 mL chloroform : 2-propanol (2:1, v/v).
3. **FFA:** Elute with 4 mL of 2 % acetic acid in diethyl ether (discard if FFA are not needed).
4. **PL:** Elute with 4 mL methanol.

If no further separation is required, points 5–9 below can be skipped. For further separation of the NL, completely dry the collected fraction to remove residues of chloroform, then redissolve the sample in 0.5 mL hexane and load it onto a new pre-conditioned SPE-column.

1. Wash the column with 4 mL of hexane to remove any cholesteryl esters.
2. TAG: Elute with 6 mL of diethyl ether: methylene chloride : hexane (1:10:89).
3. Wash the column with 4 mL of 5 % ethyl acetate in hexane to remove cholesterols.
4. DAG: Elute with 4 mL of 15 % ethyl acetate in hexane.
5. MAG: Elute with 4 mL chloroform : methanol (2:1, v/v).

Dry all the collected fractions with an inert gas, then dissolve them in 1 mL of toluene and continue to Step 3.

## Optional: gravimetry for total lipid content

We recommend quantifying total lipid content if sufficient sample biomass is available. The total-lipid content is an essential metric for many ecological analyses. In many cases, it also allows for adjusting the samples to a similar concentration range and GC peak heights, simplifying subsequent GC analysis and data processing.

It is recommended to use pre-weighed thick-walled tin cups typically used for liquid samples in Elemental Analysis (EA). We usually allocate 10 % of the sample for gravimetrical determination of total lipid content in duplicate, i.e., 2 × 50 µL of the toluene extract. As toluene has a relatively high boiling temperature, we recommend drying the tin cups overnight and determining the total lipid content the following day.

## Step 3: transmethylation

For fatty acid methyl ester (FAME) formation, it has been suggested that using sulfuric acid as a catalyst yields the best fatty acid transmethylation results [42]. Other methods described in the literature use boron trifluoride in methanol or the hydrochloride acid in methanol [43], however, we do not have experience regarding their effects on FAME *δ*^2^H and *δ*^13^C values. First, add 2 mL of 1 % H_2_SO_4_ in methanol solution to the sample in toluene, then incubate the mix for 16 h at 50 °C and let the sample cool to room temperature for 30 min. Then add 2 mL of equal normality KHCO_3_ solution (37.5 mg L ^−^ ^1^) and 3 mL of hexane. After shaking the samples, release the pressure by twisting the cap and vortex vigorously to ensure a thorough mixture of the aqueous and organic phases. Then, centrifuge at 500 *g* for 3 min. Transfer the upper organic layer into a new tube. At this point, it is critical not to transfer any aqueous phase or particles. It might be beneficial to generously leave some of the organic phase and instead add 3 mL of hexane, shake, vortex and centrifuge again, and pool the organic phases for maximal efficiency. The organic phases are dried under inert gas. This step is important because using a mixture of toluene and hexane as the solvent for injection into the GC leads to broadening peaks in the chromatogram. The samples are transferred into GC vials for analysis. If low sample volumes are required, special GC vials with low-volume glass inserts are beneficial, and the autosampler can handle volumes as low as 10–25 µL. This option is usually required when analyzing low amounts of FAME (<0.4 mg of lipid extract). Optionally, the sample volumes can be adjusted by biomass (if similar sample types were processed) or, ideally, by the total lipid content as determined gravimetrically. This way, similar peak heights are obtained in the GC chromatograms, which reduces repetitions due to peak heights falling above or below the detection range of the GC system.

It is reported that the effect of H-isotopic fractionation induced by the process of FA-methylation is usually below the detection limit of IRMS systems [44]. However, adding the methyl group might lead to a bias of FAME vs. true FA values [23]. Therefore, we recommend saving aliquots of each batch of methanol used for transmethylation to determine *δ*^2^H_MeOH_ and *δ*^13^H_MeOH_ values as described in Step 4 and to perform a methyl-group correction to the final *δ*-values as described in Step 6.

In many cases, the extracted and methylated FAME samples will be used for qFA and FA-CSIA. In this case, it is recommended first to perform the qFA, which usually requires lower sample concentrations. As a rule of thumb, if sample peaks have ideal amplitudes for GC-FID/GC–MS (i.e. they are in the middle of the calibration curves), they should be ten times more concentrated for GC-IRMS. Simultaneous measurement using splitting valves (1:10 to 1:20) is possible. However, in our experience, these setups have a high tendency for leakages, high backgrounds and non-laminar flows; therefore, we prefer performing qFA and FA-CSIA in separate runs.

## Step 4: GC-IRMS setups, general issues

For compound-specific measurements, the eluting FA are converted to H_2_ or CO_2_ gasses, on which isotope ratios can be determined by the IRMS. For carbon, the eluting FA components are combusted to CO_2_ gas in the presence of oxygen (oxidation), while for hydrogen, the components are reduced to H_2_ in the absence of oxygen (pyrolysis). These isotopes require two different reactor configurations. For some instruments (e.g., ThermoFisher), they can be mounted in parallel and toggled via valves, while for other instruments (e.g., Elementar), a complete switch of installed reactors has to be performed. The key issue to reliable IRMS measurements is a stable background. This results in two options; using an isothermal GC at high temperatures and accepting high but stable backgrounds and increased rates of column bleed, or having longer temperature-ramped runs with gentle changes in GC conditions and a return to background conditions before each new sample. These properties lead to two opposite strategies regarding GC settings, including different GC columns, for each stable isotope. The difference is mainly due to the higher carrying GC column capacity required to achieve sufficient IRMS H_2_ gas peak heights for reproducible determination of *δ*^2^H values. On the other hand, a high-capacity GC setup leads to unacceptably high and fluctuating IRMS background levels when in carbon-isotope mode. Our attempts to find a suitable compromise setup for both isotopes failed, mainly because the loss of IRMS signal intensity made the *δ*^2^H determination for trace essential FA such as EPA and DHA almost impossible.

After a FA sample entirely passes through either GC-IRMS reactor, it requires time to elute the produced gasses and return to steady-state GC and IRMS background conditions. Background conditions are ideally reached before analyzing the following sample to avoid carry-over effects. In GC-IRMS, peaks are usually broader and show more tailing than GC–MS or GC-FID in the chromatogram. Thus, good peak separation should be confirmed using isotopic reference sample mixtures. While the elution of gas from the reactors is accelerated at higher flow rates, conversion rates are higher at lower flow rates, increasing peak heights and improving reproducibility. We optimized our GC programs to find a compromise for our setup (GC Trace 1310 with a Split/Splitless Liner with Single Taper (4 × 6.3 × 78.5 mm), ConFlo IV, Delta Advantage V, all ThermoFisher Scientific). Still, slight adjustments in both temperatures of isothermal plateaus and flow rates might be necessary depending on the instrumental setup. Separation of peaks of interest is checked best using a standard mix with known components (e.g., 37-component FAME mix, 47,885-U, Supelco; Sigma-Aldrich, Bellefonte, Pennsylvania). Ideally, a return to IRMS background should be achieved. However, in practice, it is impossible to achieve this for all peaks in a sample. Still, the resolution between peaks should be at least 95 % for reliable determination of component-specific isotope composition. A possibility to determine *δ* values of low abundant peaks is the preanalytical sample concentration and/or increasing the injection volume. It is thereby recommended to enable the helium backflush during the elution time of high abundant peaks in order to prevent oversaturation of the detector and the reactor, potentially also leading to memory effects.

A large variety of different FA species containing 18 carbon atoms can be found in all types of biological samples, usually leading to a notable increase in the background in that area of the chromatogram. Accordingly, we report (below) two different GC programs for each stable-isotope setup. A shorter program is suitable for most consumer tissue and enables high throughput analysis. A second program aims for optimal peak separation for complex samples that are more time-consuming. Neither program achieves complete separation of monounsaturated FA isomers (e.g., 16:1n-7/16:1n-9; 18:1n-6/18:1n-7/18:1n-9/18:1n-12). We therefore recommend treating these isomers as a single peak and to routinely calculate the isotope value of Σ16:1 and Σ18:1, respectively. If 16:1 and 18:1 isomers shall be separated (e.g., for analysis of methanotrophic bacteria) less polar columns, such as HP-5 should be used [45].

Before performing any hydrogen stable-isotope CSIA isotopic measurements, users check and ensure there is low background of H_2_O (mass 18, Thermo: <2000 mV, Elementar: <5e-10), and Ar (mass 40; Thermo <100 mV, Elementar: <1e-11 nA) to ensure proper performance of the Nafion-Filter as well as leak-check the system. Every sample should begin and end with 2–3 reference gas pulses.

Newer instruments by Elementar enable parallel processing of FA quantification as well as carbon or hydrogen isotope measurements from fatty acids. Simultaneous MS information also enables simultaneous identification of the peaks. However, due to the different optimal parameters required for each analysis, an ideal instrument setup is almost impossible, prone to leakages and most importantly, in case of low analyt concentrations, might not produce sufficient signal for either analysis.

## Step 4a: setup for *δ*^13^C measurements

For *δ*^13^C analysis, sample peaks eluting from the GC are oxidized to CO_2_ in a micro-combustion reactor, filled with Ni, Pt and Cu wires, at a temperature of 1000  °C. After installing a new reactor, a slow heating process is required to pre-condition the reactor and obtain full oxidative capacity. This conditioning process should not be skipped, as maintaining the reactor in an oxidized state is essential to avoid low oxidation levels that lead to a significant shift in *δ*^13^C values. For Thermo™ instruments, this also involves a programmed oxidation procedure. For Elementar™ instruments, oxygenation is achieved using a low flow of oxygen through the reactor while operating. If possible, conduct pre-oxidation of the reactor before each sample sequence (1 h of O_2_ flow followed by 1 h He-flushing) and a short seed-oxidation at the end of each sample run (3 min O_2,_ followed by 3 min He-flushing).

As described above, a return to GC and IRMS and background and good peak separation is essential for accurate *δ*^13^C value determinations. Most GC column stationary phases contain significant amounts of carbon and are sensitive to column bleeding, which increases the CO_2_ background in the IRMS significantly, particularly at higher temperatures. Because the determination of *δ*^13^C is less dependent on high amounts of eluting material (usually 3–4 times less sample than for *δ*^2^H values) and more on clean peak separation, we prefer using thinner, longer polyethylene glycol columns (e.g., VF-WAXms, 60 m, 0.25 mm I.D., 0.25 µm FT). We usually operate the Thermo GC at a constant flow rate of 1.2–1.4 mL/min, which allows a maximal injection volume of 3.5 µL (Table 1; see also step 4). We found that carbon isotope values are less dependent on IRMS signal intensity. Low standard deviations were observed even at low signal amplitudes (down to 100 mV), thereby increasing the dynamic range and making fewer repeats necessary. We used two different GC programs, a simple short one if the sample contains only a few non-overlapping peaks (10–15), and an extended program for the separation of complex samples (e.g., seston, biofilm) to that enable clear separation of the peaks of interest from the background.

We recommend starting each sequence (60–100 samples) with an oxidation cycle of the reactor as described above, followed by stable-isotope standards USGS70/71/72 (see step 6) in duplicate. These FA isotope standards should be measured after 15 to 20 samples and in duplicates at the end of a batch. In Thermo instruments, a “short seed-oxidation” sequence should be performed after each sample.

## Step 4b: setup for *δ*^2^H measurements

For *δ*^2^H isotope analyses, GC resolved FAME are passed through a high-temperature (1480 °C) thermochemical carbon-conditioned (pyrolysis) open-tube ceramic reduction reactor (hydrothermal carbonization; HTC), which quantitatively pyrolyzes FAME molecules to pure H_2_ gas, with the sample carbon reduced and retained as graphite inside the reactor. Some manufacturers recommend running the reactor as low as 1200 °C to prolong the oven's life, but at the cost of 10–20 % of IRMS peak height and higher uncertainty in the *δ*^2^H values of lower amplitude peaks. We have observed incomplete conversion (high peaks of methane observed at mass 15 [CH_3_^+^]) at temperatures below 1450 °C and therefore cannot recommend running the HTC reactor at lower temperatures. The companies providing GC-IRMS instruments offer pre-fitted pyrolysis reactors, but we found that local ceramic manufacturers build high-quality reactors that can be adapted with graphite ferrules at a significantly lower cost. Since thin-walled ceramic reactors tend to crack after a few heating cycles, lower-cost tube alternatives are essential for high-throughput laboratories. For GC-^2^H—CSIA, some recommend using Cr-filled ceramic tube reactors. However, Cr reactors are only required if analyzing halogen or nitrogenous molecules [46,47] and are dunnecessary for FA-CSIA. We did not observe any advantages to using Cr reactors for FA-CSIA, on the contrary, it increased peak tailing and had a significant memory effect.

When installing a new ceramic reactor, after it reaches operating temperature, pre-conditioning is done by using 3 × 1 µL hexane injections followed by reverse He flushing for 1 h (disconnect the IRMS before performing reactor conditioning). After installing and conditioning the reactor, it is recommended to measure a series of standards, either stable-isotope standards or FAME standards, to ensure system stability (i.e., stable peak heights and isotope *δ*-values). Furthermore, the IRMS system should be tuned for optimal *δ*^2^H isotopic analyses per manufacturer specifications, including ensuring IRMS tuning to achieve low and reproducible H_3_^+^ contributions, high sensitivity, and stability testing in a continuous-flow mode by using a laboratory reference gas. We observed that H_3_^+^-factor corrections [48], automatically performed by the software, do not work well for all analytes. Late eluting analytes, such as polyunsaturated fatty acids with 20 or more carbon atoms, showed a strong peak amplitude-dependent shift in their *δ*^2^H values. To check for a valid H_3_^+^-factor correction, we measured the reference standard for each peak (e.g., 37-FAME Mix standard mentioned above) at five different concentrations and then applied an appropriate correction function (usually logarithmic in nature), if necessary.

For the Thermo Delta series, we observed H_2_ peak heights below 500 mV or peak areas below 4 Vs for the late and broader eluting >20 carbon FA, the standard deviation of analytical precision for *δ*^2^H values worsened [23]. (Peak amplitudes and areas refer to the total H_2_ ion intensity of mass 2). Moreover, the peak amplitudes should fall within the linear dynamic range of the IRMS instrument. For the Thermo Delta V series, the limit is ca. 15 V, which should not be exceeded or *δ*^2^H. Both limits correlate to the linearity of the H_3_^+^-factor correction. This means some samples may need to be measured twice if the mass fractions of FA of interest show significant peak differences, which can be either achieved by adjusting the sample amounts or changing the GC injection volumes.

**Table 1:** Recommended long and short GC programs for *δ*^13^C for complex (left) and simple (right) FA-CSIA samples for a VF-WAXms (60 m, 0.25 mm, 0.25 µm FT) or similar column.

	Rate [°C/min]	Temp [°C]	Hold [min]	Flow rate [mL / min]	Initial	Rate [°C/min]	Temp [°C]	Hold [min]	Flow rate [mL / min]
Initial		70	1	1.2			80	1	1.2
1	60.0	160	3		1	30.0	180	0	
2	2.4	176	4		2	2.6	245	16	
3	0.8	186	4						
4	2.2	202	3						
5	0.8	212	2						
6	1.5	230	0						
7	2.0	245	5						
total runtime		81 min			total runtime	42 min			

Examples can be seen in Fig. S3-S6.

Sufficient H_2_ signal is another reason for using high-capacity polyethylene glycol columns (e.g., VF-WAXms, or Supleco-2380, 30 m, 0.32 mm I.D., 1 µm FT), which can be operated at high He pressures without losing the laminar flow. We routinely inject samples at high He flow rates of 3.5 mL/min (and corresponding high column head pressures), which enable sample injection volumes of up to 8 µL without leading to back flashes. Subsequently, the He flow rate program is reduced to allow for the full quantitative conversion of FA, for which the flow rate should not exceed 1 mL/min. We observed a significant loss in signal intensity at higher flow rates. For example, a routinely used GC program can be found in Table 2. The (almost) isothermic nature of stable background conditions is also why we choose a relatively high film thickness of 1 µm, which increases the retention of the analytes and improves the separation without the need for a sophisticated temperature gradient. The total run time refers to the elution time of docosahexaenoic acid (22:6n-3, DHA), which is usually the last eluting peak of interest. If no late eluting peaks are seen in the sample, the GC sample run might be shortened.

**Table 2:** Recommended GC programs for *δ*^2^H FA-CSIA samples for a VF-WAXms (30 m, 0.32 mm, 1 µm FT) or similar column.

	Rate [ °C/min]	Temp [ °C]	Hold [min]	Flow rate [mL/min]
Initial		90	1	3
1	60.0	185	0	0.8 - 1
2	10	240/250*	as long as required	
total runtime	usually 50–70 min			

⁎ depending on GC-column max. bearable isothermic temperature. Examples can be seen in Figs. S1 and S2.

Each run sequence for *δ*^2^H measurements should start with an IRMS H_3_^+^-factor determination, followed by stable-isotope standards USGS70/71/72 (see Step 6) in duplicate. The H isotope standards should also be measured after 15 to 20 samples, and in duplicate at the end of each sequence. In our experience, no reconditioning of the reactor is required for H_2_, and samples can be continuously measured so long as no significant changes in *δ*^2^H values, or amplitudes of the standards are observed.

## Step 5: data analysis

The provided software accompanying the IRMS instruments enables automated analysis of the isotope ratio chromatograms. In most cases, the computerized analysis offers good results if the peak integration parameters have been optimized. Still, we strongly recommend manual evaluation of all isotopic CSIA peaks.

Regarding the definition or detection of CSIA peak start and end, the automated peak integration provided by the manufacturer is recommended because it allows for reproducible results between runs compared to manually tweaking peak integration. For Thermo DeltaV GC-IRMS instruments, we defined a change of 0.1 mV/s as the peak start and 0.2 mV/s as the peak end, with minimal heights as described in Step 4. One important consideration is the capillary length from the reactor to the IRMS inlet, where isotope fractionation might occur, and the chromatograms of the heavier isotopes (^2^HH, ^13^CO_2_) might show some delay compared to their lighter counterparts.

In our experience, the most critical point concerns GC-IRMS background calculations. Ricci et al. described the algorithms used for automated background evaluation [49], which we have also tested for ^2^H—CSIA [23]. Unfortunately, we did not find a setup that works for all chromatograms in all situations. Generally, we found the higher the peak amplitude, the less the *δ*-values were susceptible to an offset in the background calculations. For Thermo IRMS software (Isodat 3), we found the best results with the "individual background calculation'' algorithm, which chooses the lowest point in the chromatogram history for a defined period, and the "dynamic background'' algorithm, which averages the background over a specified period. For Elementar IRMS software (lyticOS) "ratiooffset'' and "BeforeandAfter'' showed good reproducible results. When evaluating hundreds of samples and chromatograms, the laborious manual integration of every peak requires considerable time. A reduction of this workload by (semi-)automated processes was desired. We employed automated analysis using at least three background calculation algorithms, depending on the stable isotope and sample type. These usually involved individual background calculations with two different history times (e.g., 10, 30 or 90 s) and dynamic background calculation algorithms (e.g., step width 80 s). If the standard deviation of the obtained different values is below twice the analytical precision, the mean of all values is the accepted result. Otherwise, the peak is flagged for later manual evaluation. In manual mode, we perform multiple individual background calculations, choosing past low points in the chromatogram, starting from the peak starting point and back by approx. 30 s. If the obtained values still show a relatively low standard deviation (< 10 ‰ for *δ*^2^H values and < 1 ‰ for *δ*^13^C values), the median of this series was the result. Otherwise, the sample was remeasured with a higher injection volume or sample concentration to obtain a higher peak amplitude, which usually reduces background calculation issues.

It is helpful in some cases to use an internal standard (23:0), as this reveals potential systematic errors within a GC-IRMS run (e.g., incomplete conditioning/oxidation of the reactor). We observed low standard deviation of the internal standard in multiple measurement series or good-performing reactors, and outliers were evident if a system fault occurred (e.g., extensive column bleeding, leakage, no reactor oxidation due to an empty oxygen gas bottle). It is also more helpful in assessing systematic drifts during a sequence than using certified external standards.

Upon defining a sample or standard gas peak, the IRMS software (e.g., IsoDat [ThermoScientific], lyticOS [Elementar]) applies a correction to the uncorrected *δ* values. Regarding *δ*^13^C values in EA oxygen-combustion-based systems, the Craig correction is typically used (see review in [50]), because ^17^O variations in the sample gas are negligible and FAME of both samples and standards consist mainly of C and H atoms (i.e., identical treatment during combustion and CO_2_ formation). For *δ*^2^H values, an H_3_^+^ correction (called linearity in IsoDat; see [48]) is pre-determined before each run and applied to all samples and standards. In both cases, the correction methods used should be reported in compliance with IUPAC guidelines [51].

## Optional: analysis of *δ*^2^H and *δ*^13^C of methanol

While for some studies, it is acceptable to report the stable-isotope values of the FAME (e.g. isotope labeling), for field studies, and especially studies investigating FA metabolism, comparing FA with different chain lengths, we highly recommend corrections for the addition of the methyl group, especially for the *δ*^2^H values. We found biases of up to 40 ‰ in uncorrected assays [23]. The *δ*^2^H and *δ*^13^C values of reagent methanol can be analyzed using an EA setup for liquid samples. Alternatively purified free fatty acids can be transmethylated and the difference between the free fatty acids and methylated fatty acids can be used to determine the isotopic composition of the methyl group using solid state EA. If an EA is unavailable, the stable-isotope values for methanol can be determined with a GC-IRMS using the reactor settings described above.

To improve the life-cycle time of the VF-wax columns, it is recommended to change to a column with a covalently bound stationary phase such as VF-1701 ms or DB-5 (e.g., 10 m, 0.32 mm ID, 0.5 µm FT). Because GC autosampler syringes for small volumes are costly, we routinely use a standard needle and wash it with toluene, followed by dipping the needle into an aliquot of the methanol without aspiration and immediate injection into the GC. The GC inlet is heated to 250 °C and injection is performed in split mode using a ratio of 1:5. The starting temperature of 40 °C is held for 1 min followed by an increase to 150 °C at a rate of 30 °C min^−1^. This results in two peaks with retention times depending on the column length, the first being the desired methanol and the second the toluene used for needle washing. We recommend 5–10 measurements of different aliquots, bracketed by duplicates of USGS70, USGS71, and USGS72 standards, for which GC oven temperatures have to be increased >230 °C.

## Step 6: standardization and methanol correction

The stable-isotopic composition of all FA should be reported in ‰ versus the international reference material, i.e., Vienna Pee Dee Belemit (VPDB, ^13^C:^12^C = 0.0111802 [52]) for carbon and Vienna Standard Mean Ocean Water (VSMOW, ^2^H:^1^H = 0.00015575 [52]) for hydrogen, but it should also be noted that there are inconsistencies regarding those values in the literature [53]. We recommend using three-point normalization. For FA, the FAME-C20 isotope standards USGS70 (*δ*^13^C = −30.53 ‰, *δ*^2^H = −183.9 ‰), USGS71 (*δ*^13^C = −10.5 ‰, *δ*^2^H = −4.9 ‰), and USGS72 (*δ*^13^C = −1.54 ‰, *δ*^2^H = +348.3 ‰) are suitable to cover most of the range of natural abundance samples (https://isotopes.usgs.gov/lab/referencematerials/USGS70-USGS71-USGS72.pdf). The USGS FAME reference materials are shipped as freeze-dried substances sealed under N_2_ at −20 °C. Before each measurement series, the USGS standards were freshly prepared, i.e., dissolved to a final concentration of 500 mg/L in hexane. Further reference material is available from the University of Indiana (Arndt Schimmelmann - https://hcnisotopes.earth.indiana.edu/reference-materials/materials-descriptions/fatty-acid-esters.html)

Values for *δ*^13^C are referenced to Vienna PeeDee Belemite:

$$\delta {}^{13}C_{FA}=\left(\frac{{}^{13}C/{}^{12}C_{Sample}}{{}^{13}C/{}^{12}C_{VPDB}}-1\right)$$

Values for *δ*^2^H are standardized against Vienna Standard Mean Ocean Water:

$$\delta {}^{2}H_{FA}=\left(\frac{{}^{2}H/{}^{1}H_{Sample}}{{}^{2}H/{}^{1}H_{VSMOW}}-1\right)$$

Delta values are multiplied by 1000 to show results in permil. For the introduced methyl group (C and H), it is recommended to perform "methanol corrections,'' especially for *δ*^2^H value eliminate significant biases due to the potentially large isotopic differences between the *δ*^2^H value of the methanol and the fatty acids in the sample:

$$\left(H_{n}+2\right)\;\delta^{2}H_{FAME}=\;\left(H_{n}-1\right)\;\delta^{2}H_{FA}+3\;\delta^{2}H_{Me}$$

where H_n_ is the number of H atoms for each fatty acid, *δ*^2^H_FAME_ the observed value for the FAME, *δ*^2^H_FA_ the desired value for the target fatty acid and *δ*^2^H_Me_ the previously determined value of the methanol used for transmethylation. The same formula is also be adopted for *δ*^13^C.

## Analytical performance in practice

When using the above sample and CSIA protocols, we determined on a GC Trace 1310 coupled via Conflo IV to a Delta V Advantage (ThermoScientific) a standard deviation for USGS70 of ± 2.7 ‰ and ± 0.61 ‰, for USGS71 ± 1.3 ‰ and ± 0.63 ‰, and for USGS72 ± 3.71 ‰ and ± 1.42 ‰ for *δ*^2^H and *δ*^13^C, respectively (*n* = 30 for each isotope and standard). In a test of repeated (*n* = 5) measurements of biological samples (e.g., fish, zooplankton, biofilm) we observed a mean standard deviation for 11 different FA of approx. 3–6 ‰ for *δ*^2^H and 0.4–1.2 ‰ for *δ*^13^C. In a series with 120 different samples, the internal standard (23:0) had a standard deviation of 4.8 ‰ for *δ*^2^H values and 1.2 ‰ for *δ*^13^C.

For Elementar instruments, the standard deviation of *δ*^2^H values was determined by using the FAME mix F8.4 (Arndt Schimmelmann - University of Indiana). Injection of 150 ng resulted in a peak amplitude of ∼ 0.5 nA, while injection of 500 ng resulted in an average peak amplitude of ∼3.0 nA. The standard deviations for the four FAME included ranged from 4.6 ‰ – 5.7 ‰ (*n* = 21). As an example for a biological sample, lipid extracts of macroinvertebrates were adjusted to obtain peak sizes of ∼1.5 nA for LIN and ALA. Douplicate runs of 24 samples resulted in a mean standard deviation of 3.4 ‰ and 5.0 ‰ for LIN and ALA, respectively. For *δ*^13^C values using 150 ng of FAME mix F8.3 resulted in peak amplitudes from 6.6 to 8.1 nA and standard deviations from 0.09 to 0.47‰ (*n* = 21). Repeated measurements (*n* = 5) of macroinvertebrate FAME samples resulted in standard deviations from 0.07 to 0.86 ‰ for the seven most abundant FAME exhibiting peak amplitudes from 0.21 to 35 nA.

## Ethics statements

No ethical considerations were required.

## CRediT authorship contribution statement

**Matthias Pilecky:** Conceptualization, Methodology, Writing – original draft, Validation. **Leonard I. Wassenaar:** Writing – review & editing. **Sami Taipale:** Methodology, Writing – review & editing. **Martin J. Kainz:** Resources, Writing – review & editing.

## Declaration of Competing Interest

The authors declare that they have no known competing financial interests or personal relationships that could have appeared to influence the work reported in this paper.

## Data Availability

Data will be made available on request.

## Appendix Supplementary materials

Image, application 1
