# Supplementary material for: Protocols for sample preparation and compound-specific stable-isotope analyses (δ2H, δ13C) of fatty acids in biological and environmental samples
Source: MethodsX. 2023 Jul 8;11:102283. doi: 10.1016/j.mex.2023.102283 (PMC10719507; doi:10.1016/j.mex.2023.102283)

Protocols for sample preparation and compound-specific stable-isotope analyses (δ^2H, $\delta^{13}C$) of fatty acids in biological and environmental samples.

Supplementary Material

Authors

Matthias Pilecky[1,2], Leonard I. Wassenaar[1,2], Sami Taipale[3] and Martin J. Kainz[1,2]

Affiliations

[1]WasserCluster Biologische Station Lunz, Inter-University Center for Aquatic Ecosystem Research, Dr. Carl-Kupelwieser Promenade 5, 3293 Lunz/See, Austria

[2]Research lab of Aquatic Ecosystem Research and -Health, Danube University Krems, 3500 Krems, Austria

[3]University of Jyväskylä, Department of Biological and Environmental Science, Survontie 9C, Finland

Corresponding author's email address and Twitter handle

matthias.pilecky@donau-uni.ac.at

Keywords

Carbon isotopes, deuterium, fatty acids, GC-IRMS, stable-isotopes, CSIA

Examples for ^{2}H-CSIA of Fatty acids

Figure S1. 37-FAME standard (47885-U, Supelco; Sigma-Aldrich, Bellefonte, Pennsylvania) on an VF-WAXms (30m, 0.32 mm, 1 µm FT). The retention time is given in seconds.

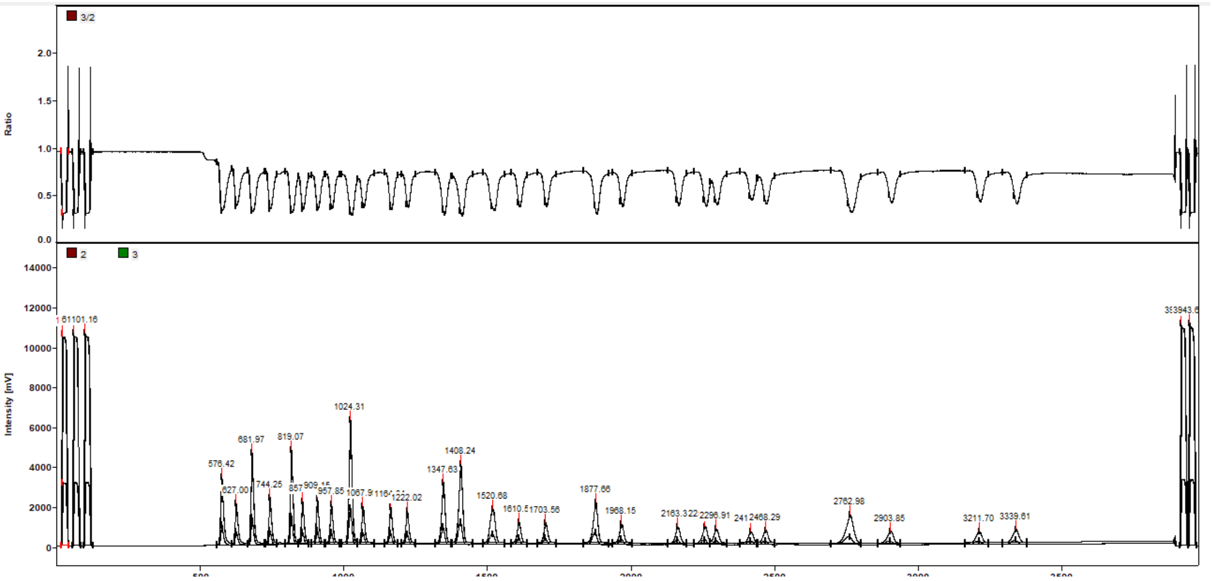

rT	Name	rT	Name	rT	Name	rT	Name
**576**	10:0	**1024**	16:0	**1703**	18:3n-3	**2762**	22:0
**627**	11:0	**1067**	16:1n-7	**1877**	20:0	**2903**	20:5n-3
**681**	12:0	**1164**	17:0	**1968**	20:1n-9	**3211**	24:0
**744**	13:0	**1222**	17:1n-7	**2163**	20:2n-6	**3339**	22:6n-3
**819**	14:0	**1347**	18:0	**2258**	20:3n-6		
**857**	14:1n-5	**1408**	18:1n-9	**2296**	21:0		
**909**	15:0	**1520**	18:2n-6	**2417**	20:4n-6		
**957**	15:1n-5	**1610**	18:3n-6	**2468**	20:3n-3		

Figure S2. ^{2}H-CSIA of fatty acids, extracted from chironomids. Note that the run has been shortened compared to the FAME standard above, because chironomids contain no unsaturated FA with > 20 C. The broader peak at 1430 sec contains all 18:1 isomers. It is recommended to report one δ^{2}H value for ∑18:1 isomers, because clear separate of the isomers while maintaining sufficient peak amplitude is difficult to achieve.

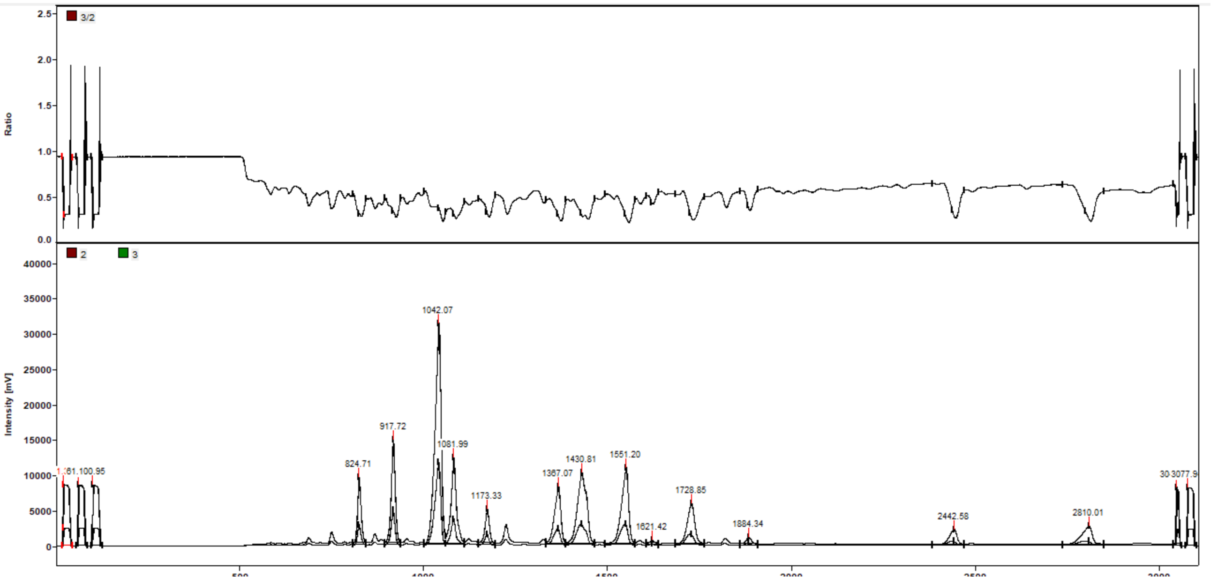

Examples for ^{13}C-CSIA of Fatty acids

Figure S3. 37-FAME standard (47885-U, Supelco; Sigma-Aldrich, Bellefonte, Pennsylvania) on an VF-WAXms (60m, 0.25 mm, 0.25 µm FT) using the longer program from table 1. The retention time is given in seconds.

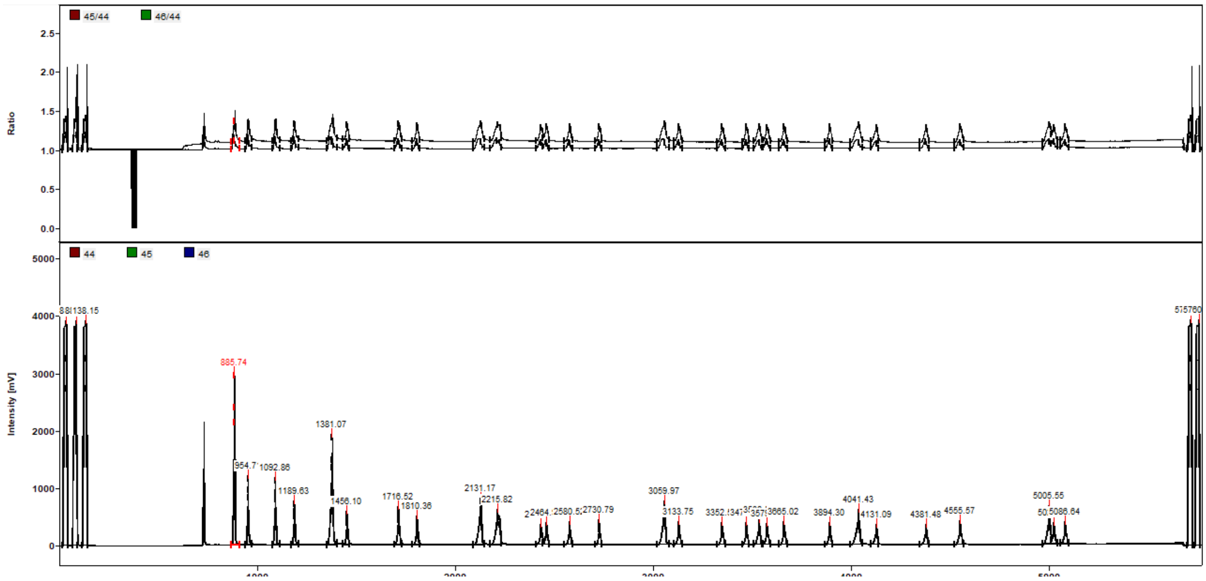

rT	Name	rT	Name	rT	Name	rT	Name
**885**	14:0	**2131**	18:0	**3474**	20:3n-6	**4555**	23:0
**954**	14:1n-5	**2215**	18:1n-9	**3538**	21:0	**5005**	24:0
**1092**	15:0	**2464**	18:2n-6*	**3579**	20:4n-6	**5027**	22:6n-3
**1189**	15:1n-5	**2580**	18:3n-6	**3665**	20:3n-3	**5086**	24:1
**1381**	16:0	**2730**	18:3n-3	**3894**	20:5n-3		
**1456**	16:1n-7	**3059**	20:0	**4041**	22:0		
**1716**	17:0	**3133**	20:1n-9	**4131**	22:1n-9		
**1810**	17:1n-7	**3352**	20:2n-6	**4381**	22:2n-6		

*the double peak is due to separation of cis and trans isomers.

Figure S4. ^{13}C-CSIA of FA of a biofilm sample brushed off of a stone from a river shore line. Especially phytoplankton samples lead to a complex chromatogram with a high number of (low amplitude) peaks, which can interfere with the determination of δ^{13}C values of peaks of interest.

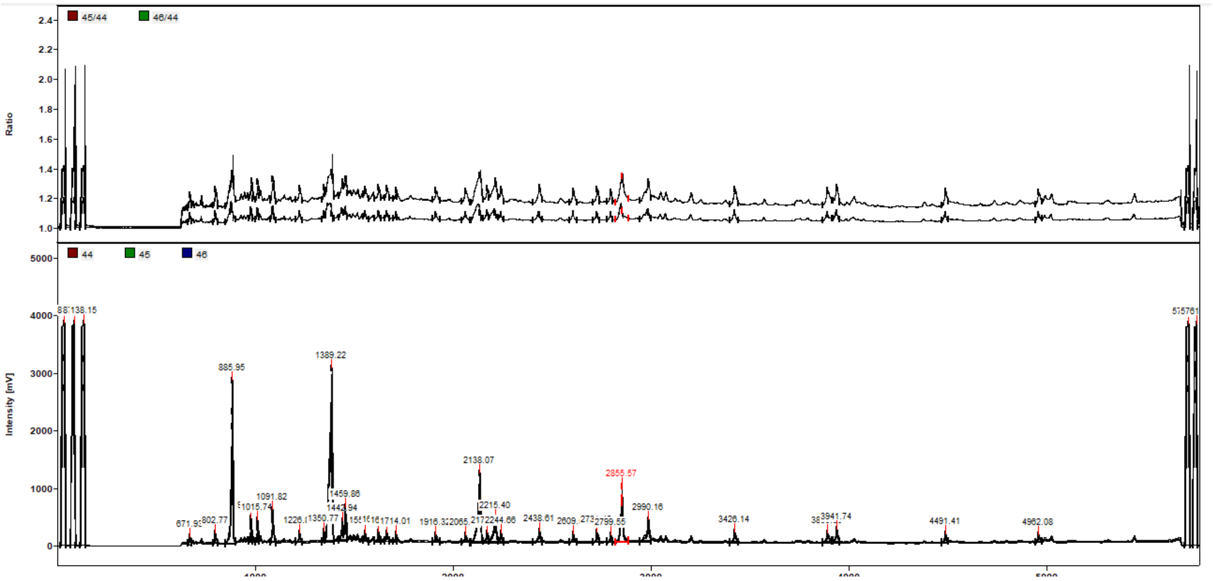

Figure S5. 37-FAME standard (47885-U, Supelco; Sigma-Aldrich, Bellefonte, Pennsylvania) on an VF-WAXms (60m, 0.25 mm, 0.25 µm FT) using the shorter program from table 1. The retention time is given in seconds.

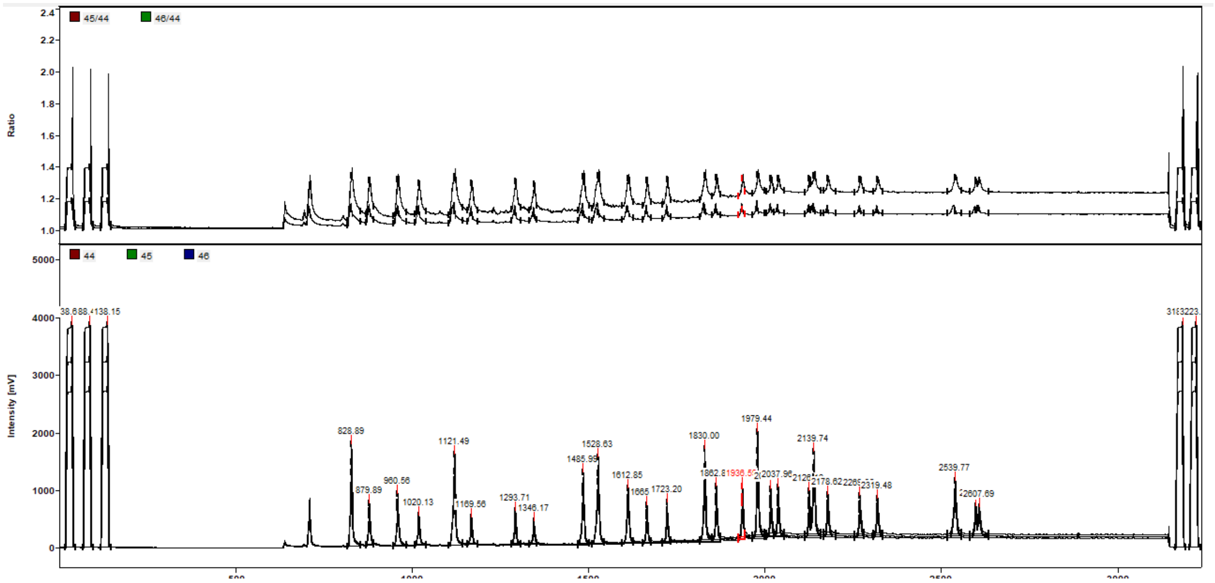

rT	Name	rT	Name	rT	Name	rT	Name
**828**	14:0	**1485**	18:0	**1979**	20:3n-6	**2319**	23:0
**879**	14:1n-5	**1528**	18:1n-9	**1979**	21:0	**2539**	24:0
**960**	15:0	**1612**	18:2n-6*	**2017**	20:4n-6	**2598**	22:6n-3
**1020**	15:1n-5	**1665**	18:3n-6	**2037**	20:3n-3	**2607**	24:1
**1121**	16:0	**1723**	18:3n-3	**2126**	20:5n-3		
**1169**	16:1n-7	**1830**	20:0	**2139**	22:0		
**1293**	17:0	**1862**	20:1n-9	**2178**	22:1n-9		
**1346**	17:1n-7	**1936**	20:2n-6	**2265**	22:2n-6		

*the double peak is due to separation of cis and trans isomers.

Figure S6. ^{13}C-CSIA of FA of daphnids isolated from a lake. Due to the lower number of interfereing peak, the shorter run program can be used.

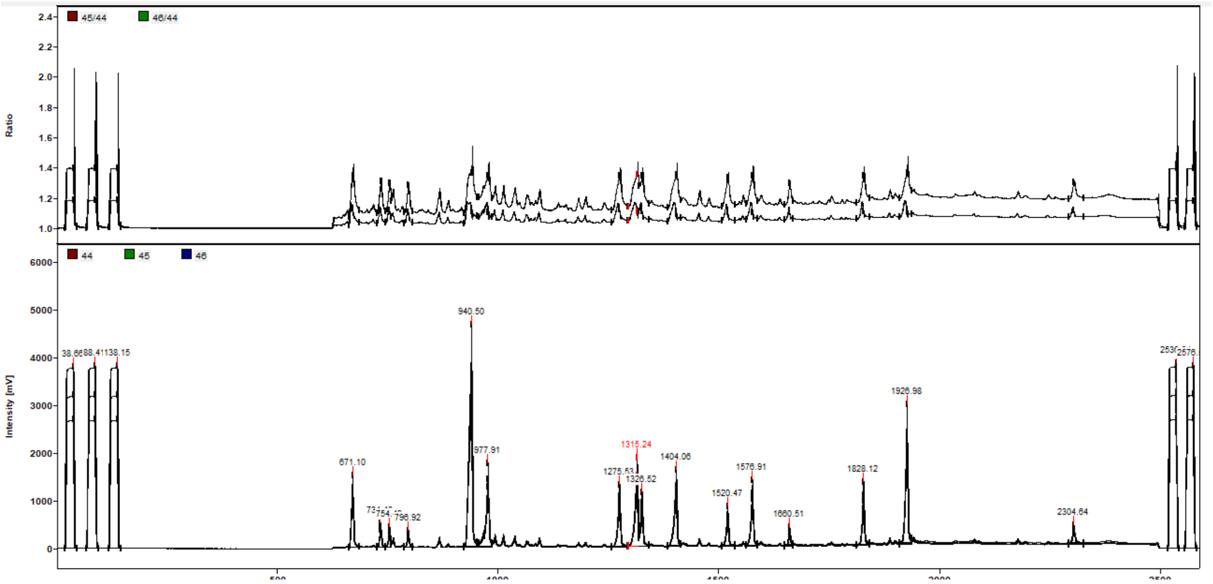

Supplement: Supplementary file 1 [file mmc1.pdf]
